# THE LANGUAGE MODELS IN HEALTHCARE AND THE ROLE OF CHATGPT: COMMENTS

**DOI:** 10.1590/0102-672020230043e1761

**Published:** 2023-09-15

**Authors:** Amnuay Kleebayoon, Viroj Wiwanitkit

**Affiliations:** 1 Academic Consultant, Research Center – Samraong, Oddar Mean Chey, Camboja; 2 Chandigarh University – Mohali, Punjab, India

**Keywords:** Health, Health facilities, Comprehensive healthcare, Artificial intelligence, Saúde, Instalações de saúde, Planejamento em saúde, Inteligência artificial

## Abstract

The introduction of chatbots has been one of the most intriguing advances in artificial intelligence. There are numerous potential uses for artificial intelligence in clinical research. However, there are also some issues that require attention. Everyone agrees that AI requires a more stable foundation and that a cutting-edge approach is necessary for AI to operate effectively.

## RESUMO

A introdução de *chatbots* foi um dos avanços mais intrigantes da inteligência artificial. Existem inúmeros usos potenciais para a inteligência artificial (IA) na pesquisa clínica. No entanto, há também outras questões que requerem atenção. Todos concordam que a IA precisa de uma base mais estável. Todos podemos concordar que uma abordagem de ponta é necessária para que a IA opere de forma eficaz.

## INTRODUCTION

Dear Editor, we found that the article on “FUTURE OF THE LANGUAGE MODELS IN HEALTHCARE: THE ROLE OF CHATGPT”, by Tustumi et al., assembles the greatest medical knowledge to develop an orientation for practice^2^.

The introduction of chatbots has been one of the most intriguing advances in artificial intelligence (AI), according to Tustumi et al.^2^. While there is no doubt that ChatGPT has the potential to transform the way healthcare is delivered, the authors came to the following conclusion: it is important to emphazise that it should not be utilized as a replacement for real healthcare experts^2^. Instead, they suggested that ChatGPT be viewed as a tool that may be employed to supplement and complement the work of these professionals, enabling them to give their patients a better treatment^2^.

There are numerous potential uses for AI in clinical research. On the other hand, there are some issues that require attention. The importance of accuracy and dependability has already been addressed. AI's potential to forecast the outcomes of clinical issues is the subject of research. It ought to be clear that using AI has some drawbacks. It should not create, analyze, or approve vital information without human review^1^. Besides, abuse may result from poor usage management strategies; for example, it might be used to automatically detect plagiarism and ghostwriting.

Undoubtedly, we all agree that AI requires a more stable foundation and that a cutting-edge approach is necessary for AI to operate effectively.
