# Supplementary material for: THE LANGUAGE MODELS IN HEALTHCARE AND THE ROLE OF CHATGPT: COMMENTS
Source: Arq Bras Cir Dig. 2023 Sep 15;36:e1761. doi: 10.1590/0102-672020230043e1761 (PMC10510369; doi:10.1590/0102-672020230043e1761)
Supplement: Related texts

# Reply: THE INDISPENSABLE HUMAN PARTICIPATION IN ARTIFICIAL INTELLIGENCE GENERATED DATA

**Authors:** Francisco Tustumi, Nelson Adami Andreollo, José Eduardo de Aguilar-Nascimento

**Affiliations:** 1 Universidade de São Paulo, Faculty of Medicine, Department of Gastroenterology – São Paulo (SP), Brazil; 2 Universidade Estadual de Campinas, Faculty of Medical Sciences, Digestive Disease Surgical Unit, Department of Surgery – Campinas (SP), Brazil; 3 Universidade Federal do Mato Grosso, Medical School, – Cuiabá (MT), Brazil; 4 Centro Universitário de Várzea, Medical School, Department of Surgery – Cuiabá (MT), Brazil.

Dear colleagues,

Thanks for thFe Letter to the Editor:

The recent announcements of artificial intelligence (AI)-based programs, including ChatGPT (OpenAi), Bard (Google), and Co-Pilot (Microsoft), raised interest in their potential impact on medical assistance and patient care. Indeed, deep learning and other AI modalities have been proposed in several fields of medicine.

Generally speaking, AI strategies provide physicians with feedback to help diagnose and treat patients. Kleebayoon and Wiwanitkit, in a letter to our previously published manuscript^3^, outlined that AI should not make vital decisions without human review. We are glad about their interest in our manuscript and completely agree with them. AI should be used as a tool for healthcare providers, not as a substitute for them.

In reality, none of the companies that announce AI-based programs claim them to be a human substitute. Human participation is indispensable in AI-generated data (Figure 1). As in any conversation, the natural (or human-like) conversation programs demand a dual interaction. In the case of chatbots, humans need to interact with the machine to create a conversation that might fulfill human interests.

**Figure 1:** The indispensable human participation in artificial intelligence generated data.

Specifically in the medical field, the healthcare provider needs to direct the machine-human chat for the benefit of the patient. Consequently, there is no doubt that the healthcare provider needs a great deal of knowledge to build a relevant clinical issue. Besides, healthcare providers must have a critical assessment capability to identify chatbot misinformation and judge if the information applies to their patients.

Numerous studies proposed using AI in computer vision to interpret images, including the use of automation in the assessment of surgical skill with machine learning algorithms^1,2^. Naturally, surgeons with no capability of interpreting AI feedback or with a deficit in surgical skills are null, even supported by these AI programs. The surgeons and all healthcare providers should recognize that they are ultimately responsible for clinical decisions and patient outcomes. The AI is only a tool.

Lastly, AI is created based on a solid amount of information. Humans are responsible for creating new data, new research manuscripts, new methods, and so on, which are the base for any AI functioning. Humans are responsible for the development and improvement of AI, making it an increasingly more valuable tool for clinical practice.

## References

[1] Lavanchy JL, Zindel J, Kirtac K, Twick I, Hosgor E, Candinas D (2021). Automation of surgical skill assessment using a three-stage machine learning algorithm. Sci Rep.

[2] Mascagni P, Vardazaryan A, Alapatt D, Urade T, Emre T, Fiorillo C (2022). Artificial intelligence for surgical safety: automatic assessment of the critical view of safety in laparoscopic cholecystectomy using deep learning. Ann Surg.

[3] Tustumi F, Andreollo NA, Aguilar-Nascimento JE (2023). Future of the language models in healthcare: the role of CHATGPT. Arq Bras Cir Dig.
